# Healthcare cost trajectories with a multidisciplinary team model: a three-year follow-up from Finnish primary and secondary care

**DOI:** 10.1080/02813432.2026.2650552

**Published:** 2026-04-11

**Authors:** Elisa Jokelin, Laura Piirainen, Mette Nissen, Erja Mustonen, Paulus Torkki

**Affiliations:** a Department of Public Health, University of Helsinki, Helsinki, Finland; b Western Uusimaa Wellbeing County, Helsinki, Finland; c Wellbeing Services County of North Savo, Kuopio, Finland; d Ministry of Social Affairs and Health, Helsinki, Finland

**Keywords:** Multidisciplinary team (MDT), primary care, secondary care, healthcare costs, chronic disease management, glycemic control

## Abstract

**Background:**

Cost pressures and limited access challenge the sustainability of Finnish primary care. Multidisciplinary team (MDT) models have been introduced, but evidence on cost and quality in Nordic settings remains limited.

**Aim:**

To describe healthcare costs and glycaemic control in primary care centres implementing an MDT model versus usual care.

**Methods:**

This quasi-experimental study included 11,124 patients from five intervention and three control health centres in Espoo, Finland. The MDT model redistributed tasks between nurses and physicians, emphasising remote consultations and proactive management. Retrospective data from primary and secondary care electronic health records (2021–2023) were analysed. Outcomes were annual per-patient costs (€) and the proportion of type 2 diabetes patients with HbA1c >53 mmol/mol. Differences over time were examined using regression models.

**Results:**

Baseline costs (2021) were similar between groups. Costs increased in both groups, but by 2023 combined per-patient costs were lower in MDT centres (4902 €) than in controls (6213 €). Primary care costs decreased slightly in intervention centres and increased in controls. Secondary care costs rose in both groups, with a steeper increase observed in control centres. Intervention centres showed a shift toward nurse-led and remote contacts with fewer physician visits. Glycaemic control remained stable in both groups. No clear differences were observed in continuity of care or avoidable hospital admissions.

**Conclusions:**

MDT implementation was associated with lower cost growth over three years without compromising glycaemic control.

## Introduction

Cost pressures in Finnish and Swedish health systems have created an urgent need for innovative care models that enable effective management of patient care at the primary care level under increasing demand [1,2]. These models aim to reduce the burden on secondary care without compromising outcomes and care quality.

In Finland, access to primary care has been a persistent challenge [3,4]. While occupational health and private providers offer on-demand services to the employed and affluent, low-income and unemployed citizens often face prolonged waiting times for appointments with primary care physicians [5]. These inequities in access have contributed to disparities in health outcomes.

To address these dual challenges of access and cost, a novel multidisciplinary team (MDT) model was implemented in Finnish primary care [6]. The model redistributes tasks, optimizes professional resources, and improves the efficiency of physician time allocation. A key feature of the intervention is its patient-centered approach, designed to ensure timely and equitable access to services.

Evidence from earlier studies suggests that integrated and multidisciplinary models of care can reduce healthcare costs while improving patient outcomes.

Various systematic reviews have reported that integrated care interventions are associated with both cost reductions and improved clinical outcomes [7,8]. Rocks et al. [7] define such interventions broadly, encompassing funding, administrative, organizational, service delivery and clinical strategies aimed at enhancing connectivity, alignment, and collaboration within and between care sectors. Jokelin et al. [6,8] focused more narrowly on primary care interventions involving professionals other than nurses or physicians added to care teams.

Further support for team-based models of care comes from three systematic reviews conducted in Canada, which found associations with reduced emergency care utilization [9,10] and decreased unplanned healthcare use among rural populations [11]. Evidence also suggests that interventions with a specific clinical focus may be more successful. Smith et al. systematic review from 2012 on care for multimorbid patients emphasized this point [12]. In a comprehensive 2019 systematic review of multicomponent interventions, most were found to have mixed effects on costs related to the Quadruple Aim goals [13,14].

Individual smaller studies have explored this issue also. For example, a social network analysis (SNA) – a method based on graph theory used to quantify collaboration, decision-making centralization and information-sharing within provider networks (e.g. among doctors and nurses) – found that highly connected primary care practices had lower hospitalization rates and healthcare spending compared to more physician-centred models [15]. A U.S. simulation study from 2015 estimated that a team-based intervention targeting uncontrolled hypertension could yield net savings of $5.8 billion over 10 years due to fewer cardiovascular events [16]. Moreover, a longitudinal study conducted in Boston (2011–2015) found that while team-based interventions reduced healthcare utilization among chronically ill patients, they paradoxically increased it among healthier individuals [17]. Nevertheless, some low-cost interventions have shown promise. For instance, a 2012 trial demonstrated that a relatively inexpensive team-based model significantly increased the number of symptom-free days for patients with chronic illness [18].

However, despite these promising findings, evidence on the cost-effectiveness of primary care MDTs remains limited – particularly within the Nordic context.

We hypothesized that the MDT model would:

1. improve access and continuity of care, leading to better health outcomes;
2. increase the number of patients treated at the primary care level, thereby reducing hospitalizations for ambulatory care-sensitive conditions and lowering overall healthcare costs; and
3. utilize remote care as an integral component to enhance accessibility and achieve cost reductions.

The primary aim of this study was to evaluate the impact of the MDT model on healthcare costs and glycemic control among patients with type 2 diabetes, coronary heart disease and/or hypertension in a Finnish primary care setting.

## Materials and methods

Table 1 gives an overview of the study design.

**Table 1.** Study design overview.

Element	Description
Design	Quasi-experimental study: intervention vs. control centers [19]
Setting	Eight primary care health centers in Espoo, Finland (population 314,150); five intervention centers (study population 6751), three control centers (study population 4393)
Participants	11,124 patients diagnosed with type 2 diabetes (ICD-10: E11; ICPC-2: T90), coronary heart disease (ICD-10: I25; ICPC-2: K74) or hypertension (ICD-10: I10; ICPC-2: K86, K87)
Intervention	Multidisciplinary team (MDT) model redistributing tasks between nurses and physicians, emphasizing remote care and proactive chronic disease management
Comparison	Usual care at three control centers without MDT implementation
Follow-up period	2021–2023
Primary outcomes	(1) Costs in primary and secondary care per patient per year (€); (2) proportion of type 2 diabetes patients with HbA1c > 53 mmol/mol
Data sources	LifeCare (primary care EHR), Apotti (hospital EHR)
Analysis	Descriptive statistics, *t*-tests or Mann–Whitney *U*-tests, and linear mixed models (*p* < 0.05)

### Description of the intervention

The implemented MDT model has been described in detail in prior work [6]. The model aims to achieve the Quadruple Aim by improving the experience of care, population health and care efficiency while reducing costs [14]. The detailed results regarding access to care and care continuity were reported in the earlier article. Access to care was consistently improved but continuity of care produced mixed results. In the usual care model, nurses determine the need for physician appointments during initial patient contacts. When physician appointments are unavailable, patients are often asked to call again later, which delays monitoring of chronic conditions.

In the MDT model, nurses and consulting physicians jointly decide the most appropriate professional to address the patient’s needs during the initial consultation, either on-site or remotely. Remote care is emphasized to enable proactive monitoring and to complete care on the same day when possible. When demand exceeds capacity, root-cause analyses are performed to identify corrective actions.

Each patient is assigned a responsible nurse, who coordinates ongoing care, including chronic disease monitoring, according to standardized protocols. The nurse consults physicians, when necessary, such as when treatment targets are not met. In the MDT model, physicians are involved earlier in the care process through structured consultative support to nurses, while nurses retain responsibility for care coordination and day-to-day patient follow-up.

The model aims at expanding systematic monitoring to a larger patient group, allowing physicians to focus on patients requiring their direct expertise, and balancing continuity, access and resource use.

#### Implementation phase and queue reset

The implementation of the MDT model included an initial transition phase during which accumulated waiting lists for non-urgent physician visits were actively reduced in the intervention centres. This queue reset was undertaken prior to the MDT model reaching a stable operational state and required temporary additional resources.

The purpose of the queue reset phase was to restore functional access to care and enable subsequent operation of the MDT model under stable conditions. Without addressing pre-existing backlogs, a reorganised care model would not have been able to operate as intended, as persistent queues would have continued to absorb available capacity. The queue reset phase and the subsequent stabilisation phase should therefore be viewed as sequential components of the overall intervention rather than as independent processes.

The queue reset involved a one-time increase in clinical capacity equivalent to approximately 20 physician working days, delivered over six weeks in one intervention health center (Iso Omena) suffering from backlog of 384 patients of which 85% were treated via telephone. Following this transition phase, care delivery shifted to a stabilised MDT-based workflow. The additional resources used for backlog reduction were temporary and do not reflect the ongoing resource requirements of the MDT model.

### Description of the setting and study population

**Figure 1.**
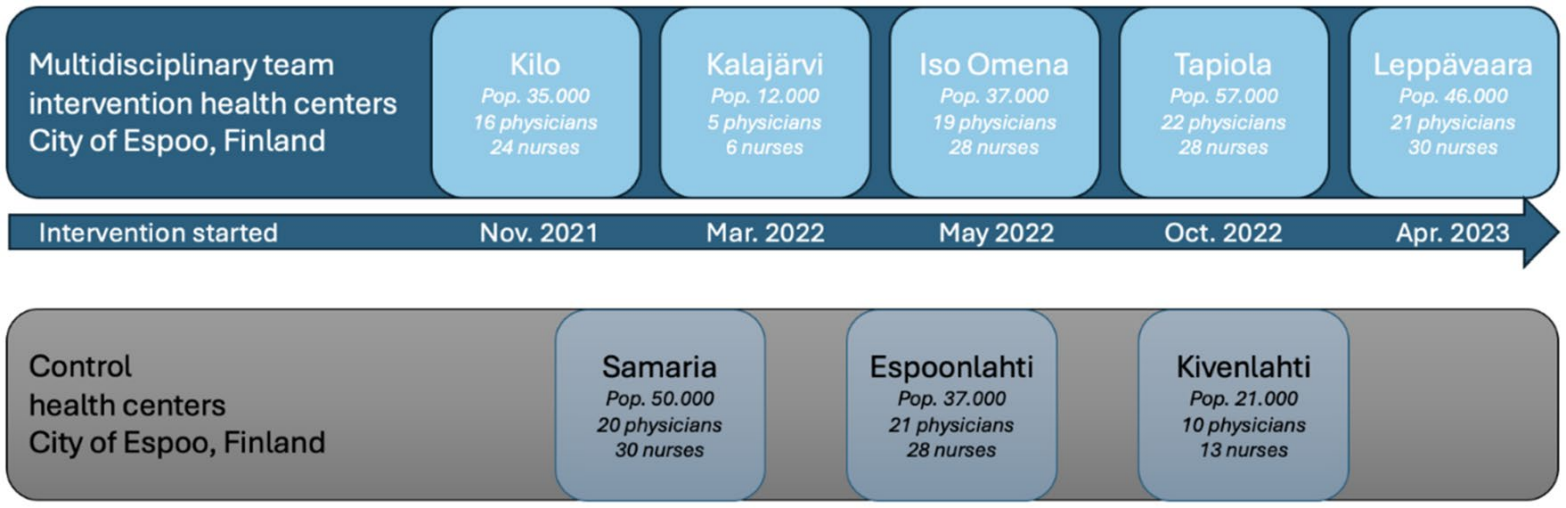
Intervention and control health centers [6].

The intervention was implemented in the city of Espoo, Finland (population 314,150). Five health centers selected by chief executive medical officers of Espoo adopted the MDT model, while three centers served as controls (Figure 1). In Finland, the health centers are serving the population of a specific catchment area. All patients registered at these centers at the end of 2023 with a diagnosis of type 2 diabetes (ICD-10: E11; ICPC-2: T90), coronary heart disease (ICD-10: I25; ICPC-2: K74) or hypertension (ICD-10: I10; ICPC-2: K86, K87) were included. The study population was defined on a population basis, including all residents in the catchment areas of the participating primary care centres, regardless of pre-existing diagnoses. Cohort membership was stable over time, with no substantial changes in population size between intervention and control centres during the follow-up period.

One control centre (Samaria) operated as a dedicated infection (COVID) unit during March 2020–September 2021. However, access to non-urgent physician care remained persistently poor across all control centres throughout the follow-up period, with the third next available appointment exceeding 90 days, as reported previously [6].

### Data collection and outcome measures

Primary care data were retrospectively collected from the LifeCare electronic health record (EHR). We recorded the number of patients with the target diagnoses, their comorbidities, and the number of patients with HbA1c levels above 53 mmol/mol. We also gathered data on nurse- and physician-led contacts (on-site visits and remote consultations) and calculated primary care costs using full-cost invoicing prices for foreigners, calculated for each welfare area, which include average laboratory and X-ray expenses (the amounts confirmed in 2024 for the Western Uusimaa Wellbeing Services County: physician on-site visit 222.60 €; nurse on-site visit 157.27 €; phone consultation 66.78 €, assistant chief medical officer, personal communication). These unit prices are based on the official, cost-based invoicing framework used within the Finnish public healthcare system and are routinely applied for inter-organisational billing. The prices incorporate average personnel, laboratory and imaging costs rather than profession-specific salary components. In accordance with this established pricing framework, the same unit cost was applied to telephone consultations irrespective of professional group. This approach reflects the underlying cost-accounting system rather than assumptions about relative professional input at the individual contact level.

Primary care costs were calculated using standardized, cost-based unit prices applied to recorded patient contacts in the EHR. These estimates reflect visit-based service delivery costs in the City of Espoo, including average personnel, laboratory and imaging costs, in accordance with the official invoicing framework used in Finnish public healthcare. The primary care cost analyses therefore represent observed service production based on documented patient contacts rather than total workforce expenditure or staffing-level costs.

Consultative physician input provided to nurses without a separately registered patient contact is not captured as a separable cost component in the EHR; however, such activity is implicitly included in the standardized visit-level unit prices through the underlying cost-allocation framework. Consequently, primary care cost results should be interpreted as reflecting differences in recorded patient-facing service delivery under a fixed pricing structure rather than detailed changes in specific internal work components or comprehensive estimates of total physician workload.

Physician and nurse contacts were classified by contact modality (in-person vs. remote) but not consistently by urgency across centres. As a result, urgent and non-urgent primary care contacts could not be reliably separated in the cost analyses but are all included. Cost estimates were based on recorded patient contacts and standardized unit costs, irrespective of whether services were delivered by permanent or temporary physicians. Staffing composition was therefore not a determinant of the cost calculations.

Hospital admission data for the same patient population were obtained from the Apotti EHR. Leppävaara health center was excluded from hospital cost analyses due to the short follow-up time (intervention launched in April 2023).

The primary outcomes were:

1. costs in primary and secondary care per patient per year (€), and
2. proportion of type 2 diabetes patients with HbA1c above 53 mmol/mol (comparison between patient’s first and last HbA1c measurement during the study period).

Cost and healthcare contact data were derived from routinely collected EHR and register sources and were available for all included patients, with negligible missingness.

For glycaemic control, HbA1c measurements were extracted from the primary care EHR. Analyses were conducted as complete-case analyses among patients with available HbA1c data. The proportion of patients with at least two HbA1c measurements and with both first and last HbA1c measurements during the study period is reported to allow assessment of data completeness (Table 2).

**Table 2.** HbA1c measurements and proportion of patients with HbA1c >53 mmol/mol by group and year.

Group	Year	Patients with HbA1c measurement, *n*	Patients with HbA1c over 53 mmol/l, *n* (%)
Intervention	2021	1793	488 (27.2%)
Control	2021	998	288 (28.9%)
Intervention	2022	1899	529 (27.9%)
Control	2022	1139	307 (27.0%)
Intervention	2023	2183	599 (27.4%)
Control	2023	1333	373 (28.0%)

Although the study population included patients with hypertension and/or coronary heart disease, disease-specific clinical quality indicators for these conditions (such as blood pressure, LDL cholesterol, smoking status or body mass index) were not sufficiently or systematically recorded in the EHR to allow valid analysis. These variables showed substantial and non-random missingness across centres and over time.

HbA1c was therefore selected as a sentinel clinical outcome due to its standardized measurement, routine use in primary care, and substantially higher completeness compared with other potential clinical indicators.

Contact analysis was done to understand the underlying mechanisms leading to costs. We analyzed physician and nurse contacts per year by type and contacts per patient.

**Figure 2.**
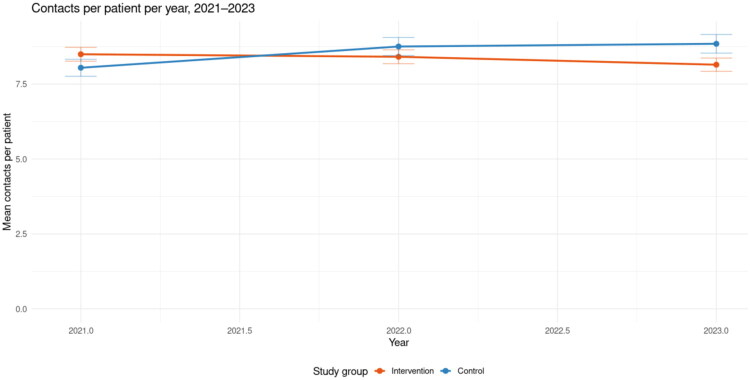
Average number of contacts per patient per year in the intervention (orange) and control (blue) groups during 2021–2023.

Secondary outcome analysis was done on Continuity of Care Index (COCI) [20] and avoidable hospital admissions for ambulatory sensitive conditions [21]. COCI was calculated monthly from the LifeCare EHR, based on the distribution of a patient’s contacts (on-site, phone or video) among physicians. The index ranges from 0 (low continuity) to 1 (all visits with the same physician). Only patients with ≥3 physician contacts within the 36-month assessment period were included. The index was calculated based on all physician contacts with the patient, including non-urgent and urgent visits as well as remote contacts, and included both permanent and temporary physicians. The pre-post analysis periods varied by health center, depending on intervention timing [6]. Data were collected from January 2021 to December 2023. Overall service use was not low in this cohort: patients had approximately 7–8 recorded contact events per year (across all professional groups, Figure 2). The number of physician contacts alone was not available as a separately extractable summary measure; total contacts are reported here only to contextualise overall utilisation and exposure to care.

COCI was calculated to investigate whether the intervention had an impact on continuity as this is an important aspect of chronic illness care. Avoidable hospital admissions served as an additional quality indicator for the intervention as well. They were defined using diagnosis-based criteria according to the Finnish national classification of potentially avoidable hospitalisations [21]. Included conditions comprised selected vaccine-preventable infections (e.g. influenza and pneumonia), chronic conditions (such as diabetes complications, asthma, chronic obstructive pulmonary disease, heart failure, hypertension and anaemia) and acute conditions (including urinary tract infections, skin and soft tissue infections, and selected dental conditions), identified using ICD-10 codes.

### Statistical analysis

We used a quasi-experimental design comparing intervention centers with control centers. Five intervention centers were compared (1) before and after the intervention and (2) with three control centers. Normality of distributions was tested using the Shapiro–Wilk test. Group comparisons were performed with *t*-tests (normal distribution) or Mann–Whitney’s *U*-tests (non-normal distribution).

Descriptive statistics were used to summarize annual per-patient costs and health care contacts. Differences between intervention and control groups over time were assessed using regression models including group, year and their interaction. Costs (primary, secondary and combined) were analyzed with linear regression and cluster-robust standard errors. For health care contacts, both ordinary least squares and linear mixed-effects models with patient-level random intercepts were applied. All tests were two-sided with *p* < 0.05 considered statistically significant. Analyses were performed using R (version 4.2.2; R Foundation for Statistical Computing, Vienna, Austria).

The difference-in-differences analyses compare changes over time between intervention and control centres. However, the intervention centres underwent an initial transition phase that included active reduction of accumulated waiting lists prior to stabilisation of the MDT model. In one intervention centre, this transition phase involved a one-time, time-limited increase in clinical capacity equivalent to approximately 20 physician working days.

As a result, the pre-intervention period may not fully represent a steady-state baseline across all intervention centres. The difference-in-differences estimates should therefore be interpreted as capturing changes associated with the transition to and subsequent operation of the MDT-based care model, rather than isolating a single discrete causal mechanism attributable solely to the MDT workflow reorganisation.

The analyses are based on year-by-group interaction terms rather than time since implementation. Consequently, the models estimate year-specific differences between intervention and control groups relative to baseline, rather than explicit changes in post-intervention slopes. The observed temporal pattern should therefore be interpreted as reflecting cumulative and persistent differences associated with the transition to and operation of the MDT-based care model, rather than as evidence of dynamic year-by-year changes in outcome trajectories attributable to a single intervention component. We did not conduct formal sensitivity or falsification analyses such as lead–lag tests, event-study specifications or interrupted time series analyses. The purpose of the analyses was not to distinguish short-term level changes at implementation from longer-term trend changes, but to assess whether differences between intervention and control centres persisted and accumulated over time following a system-level reorganisation of care.

### Ethics

This study was approved by the Ethical Committee of the University of Helsinki in January 2022. As this was a retrospective register-based study, no individual informed consent was required under Finnish legislation. Patient data were analyzed in a pseudonymized format in accordance with the EU General Data Protection Regulation (GDPR) and local data security protocols.

The intervention was implemented as part of routine care, without additional burden or risk to patients. Particular attention was paid to ensuring equitable access to care, especially for vulnerable patient groups. The involvement of one author in the development of the MDT model did not influence data collection, analysis or reporting.

## Results

### Descriptive characteristics of the study population during the follow-up period

Study populations were largely comparable between the intervention (*n* = 6731) and control (*n* = 4393) groups (Table 3). The only statistically significant difference was age, with the intervention group being older (*p* < 0.001).

**Table 3.** Descriptive characteristics of the study population during the follow-up period.

Characteristic	Intervention (*n* = 6731)	Control (*n* = 4393)	*p* Value
Age, mean (SD)	59.1 (9.5)	58.3 (9.8)	**<0.001**
Female, *n* (%)	3 352 (49.8)	2 223 (50.6)	0.418
Type 2 diabetes, *n* (%)^a^	3 165 (73.5)	1 998 (72.5)	0.393
Hypertension, *n* (%)^a^	4 488 (80.7)	3 078 (80.7)	0.951
Coronary heart disease, *n* (%)^a^	755 (11.2)	438 (10.7)	0.599
Number of diagnoses, *n* (%)			0.121
0^b^	40 (0.6)	20 (0.5)	
1	5 439 (80.8)	3 483 (79.3)	
2	1 187 (17.6)	839 (19.1)	
3	65 (1.0)	51 (1.2)	

Diagnoses and comorbidities are based on routinely registered diagnoses recorded during the follow-up period. These characteristics describe the observed case mix over time and are not intended to represent pre-intervention baseline morbidity. Percentages indicate the proportion of patients with at least one recorded diagnosis in the specified category during the follow-up period. Bold values indicate statistically significant differences between intervention and control groups (*p* < 0.05).

^a^ The percentages can be above 100% because of multiple diagnoses in the same person.

^b^ The presence of patients with zero recorded ICD-10 diagnoses reflects the inclusion of individuals whose care during the observation period was delivered exclusively by nurses, with encounters documented using ICPC-2 codes.

### Combined primary and secondary care costs

**Figure 3.**
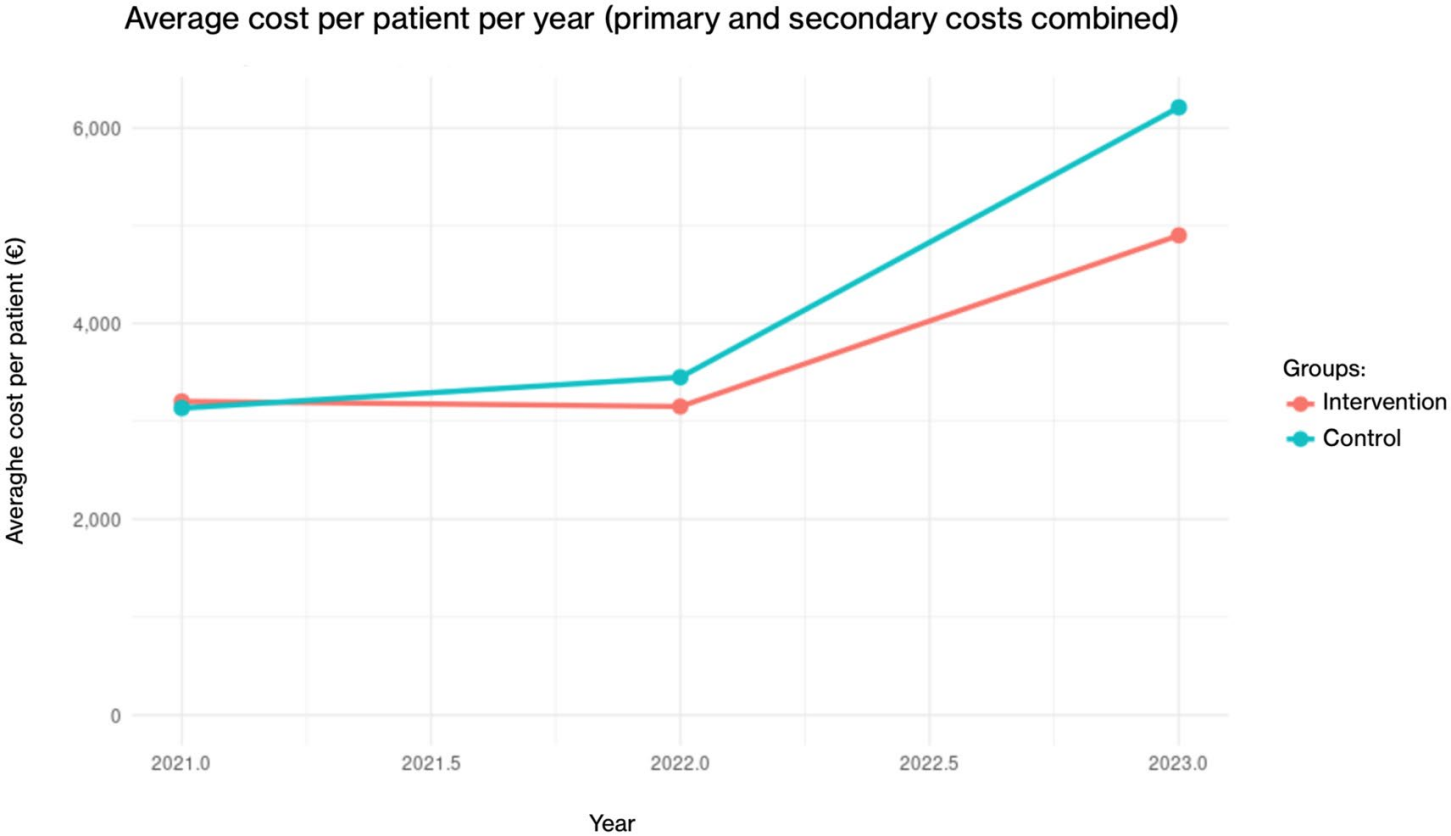
Average annual per-patient costs (primary care + secondary care combined) in the intervention and control groups during 2021–2023.

Mean annual per-patient costs (primary care + secondary care combined) increased in both groups during the follow-up period (Figure 3). At baseline (2021), total costs were comparable between the intervention and control groups (3202 € vs. 3133 €, respectively). By 2023, costs had increased in both groups, reaching 4902 € in the intervention group and 6213 € in the control group.

For combined costs (primary and secondary care), the linear regression model showed a significant annual increase in the intervention group of 563 € per patient (*p* < 0.001). The control group exhibited a significantly steeper annual increase, as indicated by the positive group-by-year interaction (+918 € per patient per year, *p* < 0.001). The faster growth rate resulted in 1311 € higher per-patient costs by the end of follow-up (*p* < 0.001).

### Primary and secondary care costs separately

**Figure 4.**
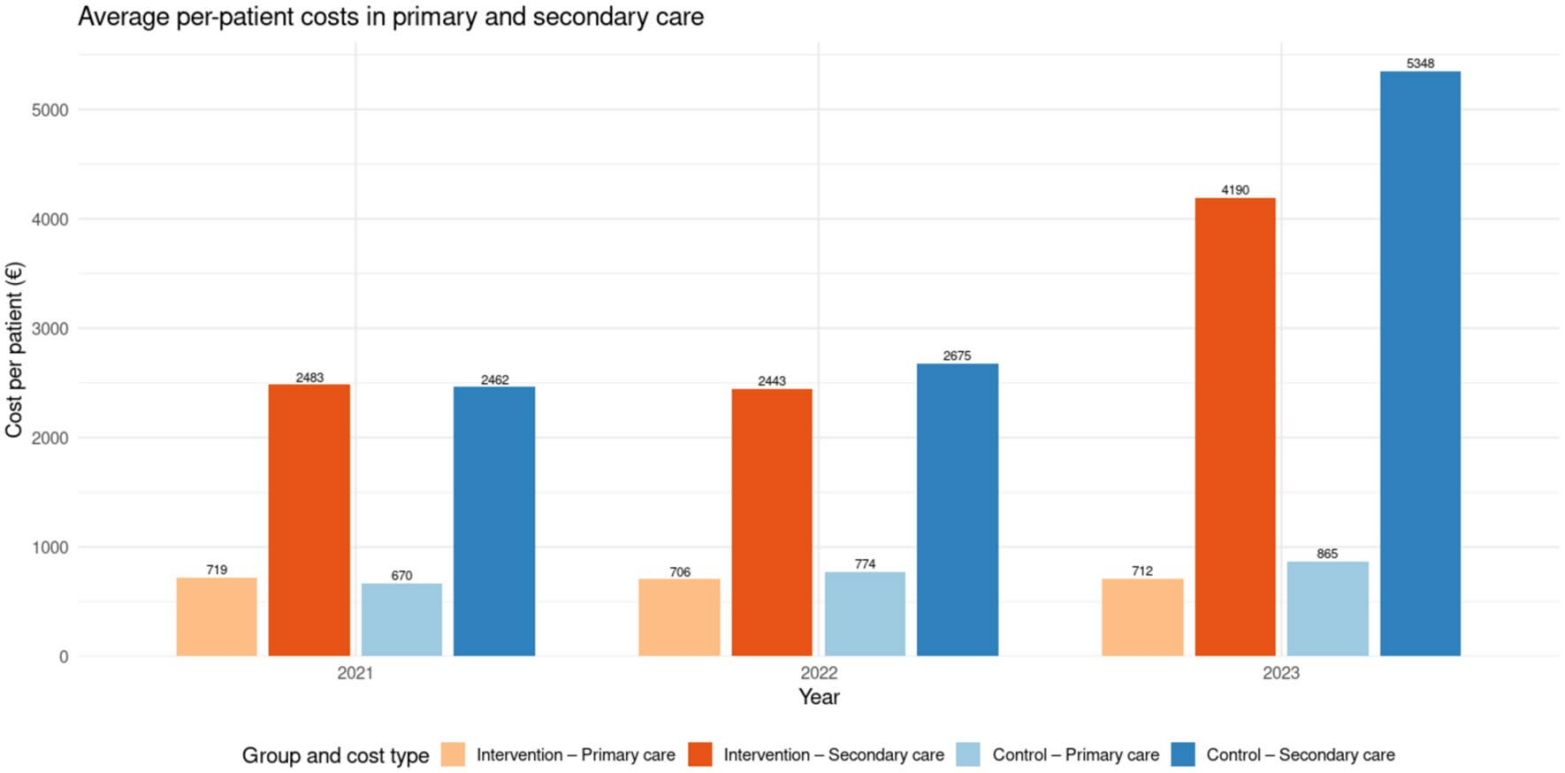
Average annual per-patient costs in primary and secondary care for the intervention and control groups, 2021–2023.

Figure 4 shows the mean annual per-patient costs in primary and secondary care for the intervention and control groups during 2021–2023. At baseline (2021), there were no statistically significant differences in costs: primary care costs were 719 € in the intervention group and 670 € in the control group, while secondary care costs were 2483 € and 2462 €, respectively.

In 2022, primary care costs remained largely unchanged in the intervention group (706 €) but increased modestly in the control group (774 €). Secondary care costs also grew more steeply in the control group (2675 €) than in the intervention group (2443 €).

By 2023, a clear divergence was observed. Primary care costs were relatively similar between the groups (712 € vs. 865 €), whereas secondary care costs increased more in the control group (5348 €) than in the intervention group (4190 €) (*p* = 0.0028).

Over time, primary care costs in the intervention group showed a modest annual decline whereas costs in the control group increased significantly faster (+104 € per patient per year, *p* < 0.001). In secondary care, costs in the intervention group rose annually by 602 € per patient (*p* < 0.001), but the increase was significantly steeper in the control group (+815 € per patient per year, *p* = 0.0028). By 2023, this divergence resulted in higher secondary care costs in the control group (5348 €) compared with the intervention group (4190 €) (*p* = 0.0028) (Table 4).

**Table 4.** Average annual per-patient costs (€) in primary and secondary care, and combined costs, in the intervention and control groups, 2021–2023.

Year	Group	Primary care (€)	Secondary care (€)	Combined (€)
2021	Intervention	719	2483	3202
2021	Control	670	2462	3133
2022	Intervention	706	2443	3149
2022	Control	774	2675	3448
2023	Intervention	712	4190	4902
2023	Control	865	5348	6213
	Regression results			
	Primary care	+104 per patient per year	<0.001	
	Secondary care	+815 per patient per year	0.0028	
	Combined costs	+918 per patient per year	<0.001	

Linear regression models (group × year interaction) are shown below; positive estimates indicate a steeper annual increase in costs in the control group compared with the intervention group.

### Glycemic control

No statistically significant differences were found between groups in the proportion of patients with HbA1c levels above 53 mmol/mol over time (*p* = 0.261, comparison between the first and last measure per patient during the follow-up time) (Table 2). In 2021, proportion of patients above target was 27.2% in intervention group and 28.9% in control group. By 2023, the proportion of patients above target had risen to 27.4% in intervention group and decreased to 28% in the control group.

### Underlying factors behind the cost changes: contact types

**Figure 5.**
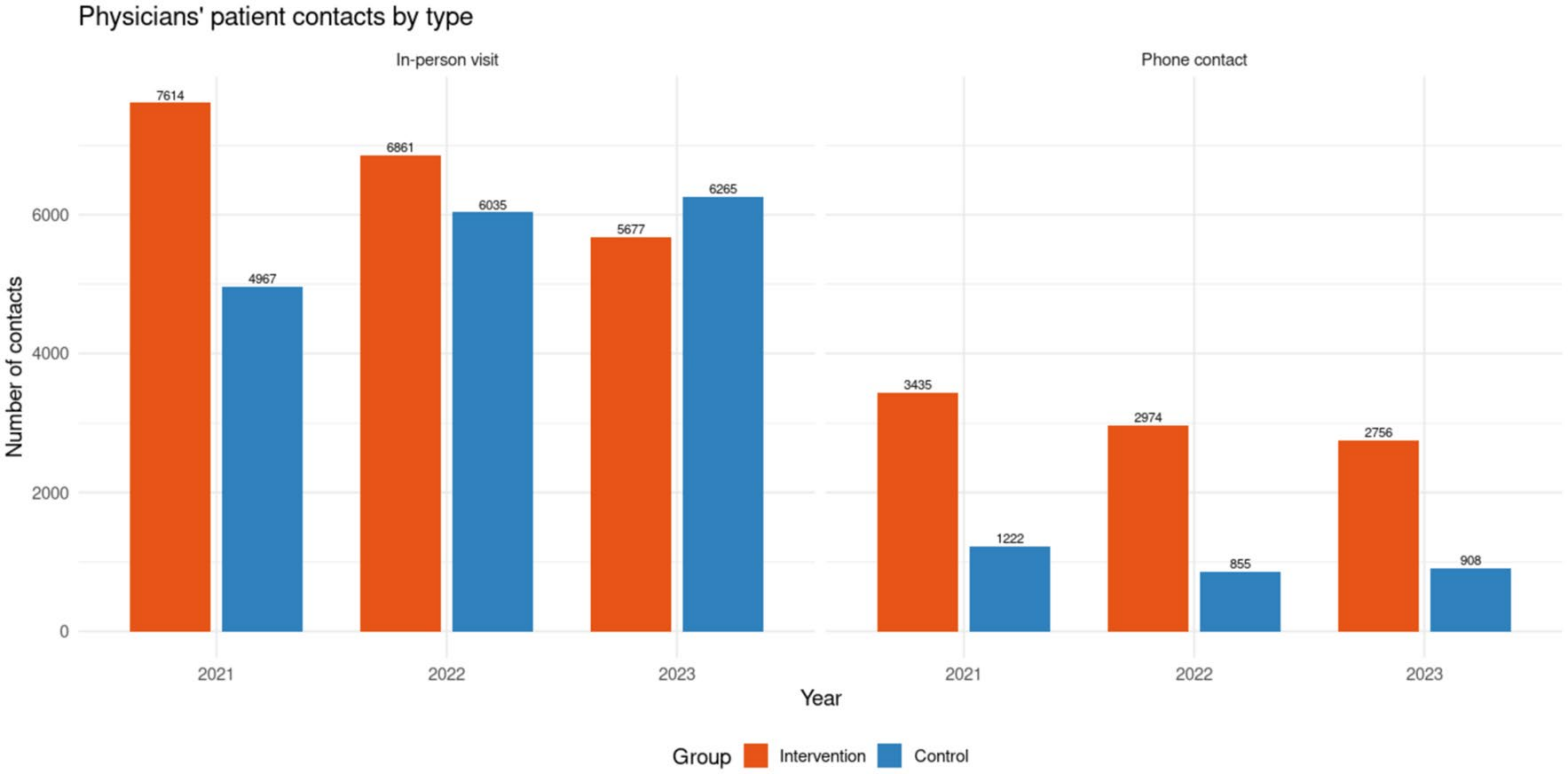
Physicians’ patient contacts by type in the intervention and control groups during 2021–2023.

Physicians’ contacts declined over time in intervention group (Figure 5). Phone contacts decreased in the intervention group from 3435 in 2021 to 2756 in 2023 (−20%), and in the control group from 1222 to 908 (−26%). In-person visits also declined in the intervention group (7614–5677, −25%) but increased in the control group (4967–6265, +26%). Regression analysis confirmed these trends: baseline contact rates were significantly lower in the control group compared with the intervention group (−354, *p* < 0.001). Over time, physician contacts declined significantly in the intervention group (−0.22 per patient per year, *p* < 0.001), while the decline was less pronounced in the control group, as shown by the positive group × year interaction (+0.18, *p* < 0.001). Absolute levels of physician contacts per patient-year are not reported separately, as physician contacts could not be reliably extracted as a standalone utilisation metric in the final dataset; changes are therefore reported as differences per patient-year.

**Figure 6.**
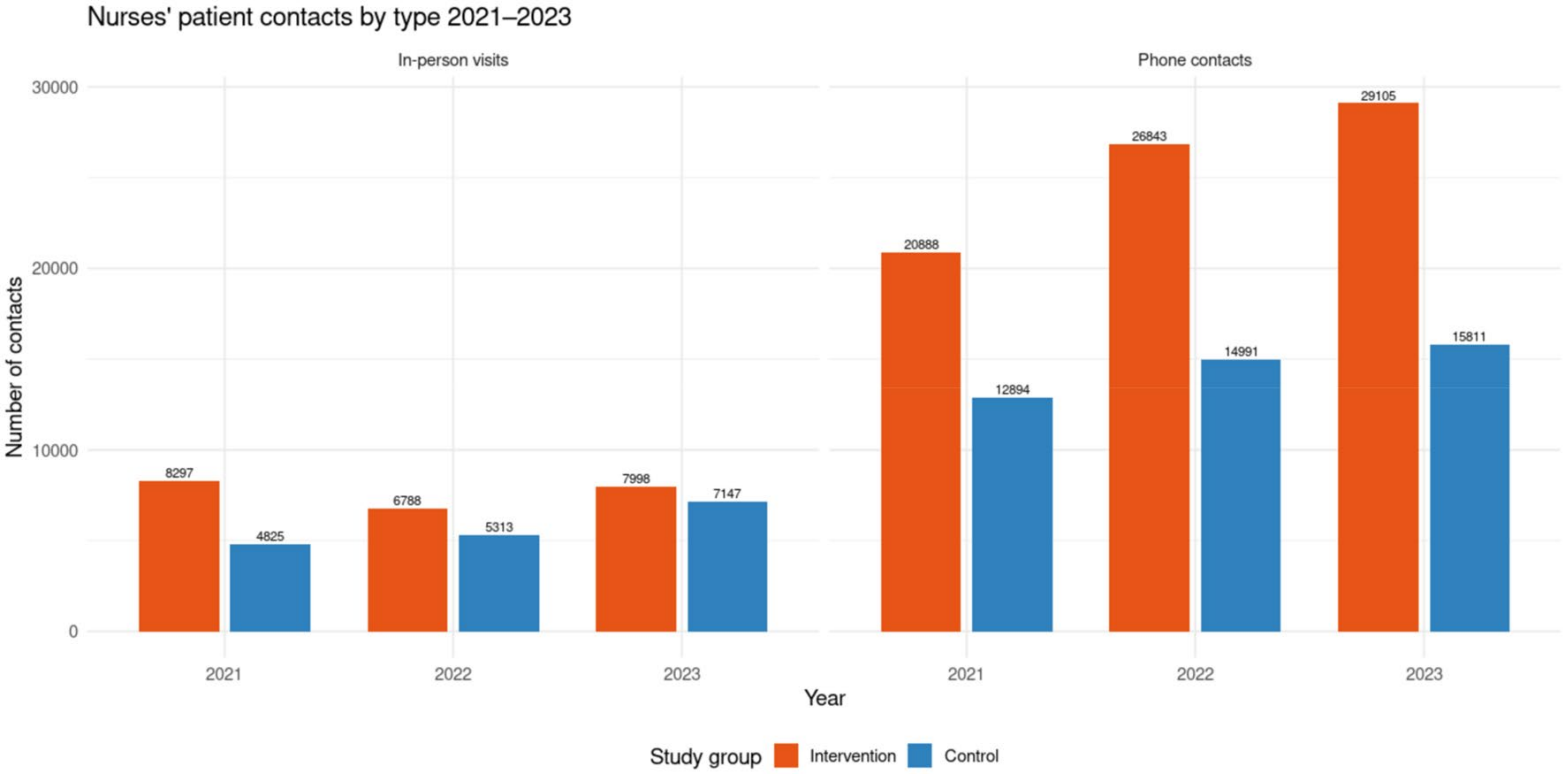
Nurses’ in-person visits and phone contacts in the intervention and control groups during 2021–2023.

In contrast, nurses’ contacts increased in both groups (Figure 6). Phone contacts rose from 20,888 in 2021 to 29,105 in 2023 in the intervention group (+39%), compared with an increase from 12,894 to 15,811 in the control group (+23%). In-person visits also decreased in the intervention group (from 8297 to 7998, −4%) while they increased in the control group (from 4825 to 7147, +48%). Statistical analysis indicated no significant baseline difference between groups (*p* = 0.91). Nurse contacts increased significantly in the intervention group over time (+0.30 per patient per year, *p* < 0.001), but the group × year interaction was not significant (+0.009, *p* = 0.91), suggesting that the upward trend was similar in both groups.

For nurses’ contacts, distinct patterns emerged between phone contacts and in-person visits. At baseline, phone contacts were higher in the control group than in the intervention group, corresponding to a group-level difference of +548 contacts (*p* < 0.001). Over time, phone contacts increased significantly in the intervention group (+0.40 per patient per year, *p* < 0.001), while the increase was slower in the control group, as shown by the negative group × year interaction (−0.27, *p* < 0.001).

In contrast, baseline levels of in-person visits were lower in the control group compared with the intervention group, corresponding to a group-level difference of −498 contacts, *p* < 0.001. No significant annual change was observed in the intervention group (*p* = 0.20). However, the group × year interaction was positive (+0.25, *p* < 0.001), indicating that in-person visits increased significantly faster in the control group than in the intervention group.

For physicians’ contacts, both phone and in-person visits declined over time, but the patterns differed between groups. For phone contacts, no significant baseline difference was observed between the groups (*p* = 0.85). Contacts declined significantly in the intervention group (−0.07 contacts per patient per year, *p* = 0.0014), with a similar downward trend in the control group, as indicated by the non-significant interaction (*p* = 0.84).

In contrast, for in-person visits, baseline levels were significantly lower in the control group compared with the intervention group corresponding to a group-level difference of −411 contacts (*p* < 0.001). Visits declined significantly in the intervention group over time (−0.15 per patient per year, *p* < 0.001). The group × year interaction was positive (+0.20, *p* < 0.001), indicating that the decline was significantly steeper in the intervention group than in the control group.

#### Contacts per patient

The mean number of contacts per patient per year decreased in the intervention group and increased in the control group during the study period (Figure 2, Table 5).

**Table 5.** Mixed-effects regression results for physicians’, nurses’ and total contacts per patient per year.

Outcome	Variable	Estimate	Std. error	*t* value	*p* Value
Physicians – phone	Intercept	149	46.1	3.23	**0.0013**
	Control (vs. intervention)	13.3	69.4	0.19	0.85
	Year	−0.07	0.023	−3.19	**0.0014**
	Group × year	−0.007	0.034	−0.2	0.84
Physicians – in-person	Intercept	305	34.6	8.82	**<0.001**
	Control (vs. intervention)	−411	57.2	−7.18	**<0.001**
	Year	−0.15	0.017	−8.76	**<0.001**
	Group × year	0.20	0.028	7.18	**<0.001**
Nurses – phone	Intercept	−808	72.7	−11.1	**<0.001**
	Control (vs. intervention)	548	115.3	4.75	**<0.001**
	Year	0.40	0.036	11.2	**<0.001**
	Group × year	−0.27	0.057	−4.76	**<0.001**
Nurses – in-person	Intercept	102	78.4	1.3	0.19
	Control (vs. intervention)	−498	118	−4.23	**<0.001**
	Year	−0.05	0.039	−1.27	0.20
	Group × year	0.25	0.058	4.23	**<0.001**
All contacts	Intercept	260	121	2.15	**0.032**
	Control (vs. intervention)	−126	19.3	−6.54	**<0.001**
	Year	−0.12	0.060	−2.08	**0.037**
	Group × year	+0.62	0.095	6.54	**<0.001**

Estimates indicate baseline differences, annual changes and group-by-year interactions between intervention and control groups. Bold values indicate statistically significant differences between intervention and control groups (*p* < 0.05).

Mixed-effects regression confirmed these patterns. At baseline, overall contact levels were lower in the control group, corresponding to a group-level difference of −126 contacts (*p* < 0.001). A modest annual decline was observed in the intervention group (−0.12 contacts per patient per year, *p* = 0.037). However, the positive group × year interaction (+0.62, *p* < 0.001) indicated that contacts increased significantly faster in the control group than in the intervention group.

### Secondary analyses

No statistically significant differences were observed in the COCI or hospital admissions for ambulatory care-sensitive conditions between groups. Absolute levels of physician continuity were low in both groups throughout follow-up. Mean COCI values ranged from 0.13 to 0.18 in the intervention group and from 0.09 to 0.13 in the control group. From 2021 to 2023, the increase in COCI was +0.018 in the intervention group compared to the control group (difference-in-differences, *p* = 0.077).

Only small percentage of patients in either group had hospital admissions for ambulatory care-sensitive conditions: in 2021, 0.50% in the intervention group compared to 0.44% in control group. In 2023, the percentages were similar: intervention group 0.51% and control group 0.44%.

## Discussion

### Main findings

In this quasi-experimental study, the implementation of a MDT model in Finnish primary care was associated with a slower increase in combined primary and secondary care costs without compromising glycaemic control or other indicators of care quality. Visit-based primary care costs decreased slightly with the intervention while control group’s primary care costs increased significantly. Secondary care costs increased in both groups, but this development was significantly slower in the intervention group.

Changes in visit-based primary care costs were accompanied by a shift in service delivery: more patient contacts were managed by nurses, and phone consultations increased while in-person visits declined. Physicians’ in-person visits also decreased slightly, likely reflecting a reallocation of physician time toward consulting with nurses to determine appropriate patient management. Over time, total patient contacts per capita decreased in intervention centers, which may indicate changes in care processes and utilisation patterns following the reorganisation of care. These changes are consistent with a more proactive and coordinated approach to primary care delivery. We further note that the slower increase in secondary care costs occurred in parallel with improved access to primary care at intervention sites reported in our prior work. While specific secondary care pathways could not be disentangled in the present study, the observed divergence in secondary care cost trajectories represents a robust system-level finding. The observed changes in utilisation and costs are likely influenced by multiple interacting factors. Improved access to non-urgent care following the reduction of accumulated waiting lists is one important determinant, as restored access alters patient flows and service use patterns. In the present study, the processes used to address accumulated queues differed between intervention centres.

In one centre (Iso Omena), backlog reduction involved a one-time, time-limited increase in capacity equivalent to approximately 20 physician working days. In another centre (Kilo), queues were addressed during the initial phase of MDT implementation through reorganisation of team-based workflows without additional staffing resources. In the remaining centres (Tapiola and Leppävaara), waiting lists had been reduced prior to MDT implementation through internal measures. Following these transition phases, all intervention centres operated under a stabilised MDT-based workflow.

At the same time, changes in care processes associated with the MDT model – including task redistribution, increased use of remote contacts and structured care coordination – are likely to have influenced how care demand was managed once access had been restored. In this context, improved access should not be viewed as an external confounder but as a necessary precondition for the MDT model to function as intended. A care model that continuously accumulates queues cannot be considered operationally stable; both backlog reduction and subsequent workflow reorganisation are required to achieve sustained changes in service delivery.

Although one control centre operated as a dedicated infection unit during part of the study period, this organisational role does not alter the central contrast of the study. The comparison is not between specific organisational models within primary care, but between settings with persistently poor access to non-urgent care and settings where access was restored and maintained.

In the control group, access to primary care remained markedly constrained throughout follow-up, irrespective of organisational arrangements. The observed divergence in secondary care cost trajectories is therefore interpreted primarily in relation to differences in primary care access rather than differences in specific service configurations within the control group.

Physician continuity was low in both groups, reflecting a broader post-pandemic primary care context characterised by workforce shortages, reliance on temporary physicians and organisational instability. Under these conditions, large improvements in physician-level relational continuity are unlikely in the short term.

Importantly, improved access and increased service availability associated with the MDT model did not lead to further deterioration in physician continuity, despite changes in care organisation and contact patterns. Physician COCI may also be insufficiently sensitive to capture continuity mechanisms central to the MDT model, where care coordination and relational continuity are primarily provided by designated nurses rather than by repeated physician encounters. In this context of generally low physician continuity, we acknowledge that COCI values have limited capacity to discriminate differences in broader quality of care between organisational settings.

The intervention evaluated in this study represents a system-level reorganisation of primary care rather than a single, isolated change. The implementation involved multiple concurrent elements, including backlog reduction to restore access, introduction of MDT-based workflows, changes in task allocation, increased use of remote contacts, and adaptations in leadership and operational management.

Within such a complex system change, it is not possible to disentangle the individual contributions of specific components (e.g. MDT structure, Lean-inspired process redesign or management practices) using routine register data alone. The results should therefore be interpreted as reflecting the combined effects of these interacting elements on service use and cost trajectories, rather than ascribing observed temporal patterns to a single causal mechanism.

A key question in the interpretation of the findings is the relative contribution of short-term transition effects (such as backlog reduction and access restoration) versus ongoing effects of the MDT-based care model. In principle, some operational indicators – such as telephone access metrics – may exhibit pronounced short-term changes during early implementation phases, often worsening initially before improving as new workflows stabilise. However, such indicators are not routinely or consistently available in a form that would allow robust time-series analysis across centres.

Importantly, the primary contribution of the present study is not to isolate short-term implementation effects, but to demonstrate that improvements associated with the reorganisation of care were sustained over a multi-year follow-up. While many short-term interventions may temporarily improve access or utilisation, a central question for health systems is whether such gains persist. The MDT model evaluated here was associated with a sustained divergence in cost and utilisation trajectories over three years, suggesting that the improved level of performance was maintained rather than reverting once initial transition effects dissipated.

### Comparison with previous studies

Unlike most evaluations that report either primary-care spending or hospital use in isolation, we quantified combined primary + secondary costs per patient and showed a divergent trajectory by care model. Our findings are consistent with previous research showing that integrated and team-based care models can improve cost-efficiency while maintaining or improving care quality. Kuo et al. reported that a higher degree of connectedness among primary care team members was associated with lower spending, which they attributed to better care coordination, faster information sharing, timely communication and more effective service delivery [15].

Similarly, Rocks et al. found in their systematic review that integrated care interventions targeting disease management were particularly effective in reducing costs and improving outcomes [7]. Our results align with this evidence, as the MDT model emphasized nurse coordination and proactive management of chronic diseases. This systematic approach likely contributed to the observed reductions in costs and improved resource use. Furthermore, Jokelin et al. have reported comparable findings for disease management programs in chronic illness patients, supporting the relevance of such models in primary care [8].

### Implications for policy and systems

We assessed clinical quality (e.g. glycaemic control) and other process indicators alongside costs. The MDT model did not compromise quality, addressing the common concern that cost containment erodes outcomes. These findings are highly relevant in the context of ongoing health system reforms in Finland, which aim to enhance continuity of care, reduce fragmentation and costs, and improve access. The MDT model supports these objectives by leveraging the skills of the wider healthcare team around the physician, thereby advancing the goals of the Quadruple Aim without over-relying on physician-centred care. We linked cost changes to task redistribution (nurse-coordinated, remote contacts increased; physician in-person decreased) and access/continuity (named nurse), offering a plausible pathway for the observed slowdown in secondary-care costs. This approach optimizes task distribution and resource use, and its scalable design makes it a promising strategy for addressing persistent challenges in both Finnish and broader Nordic healthcare systems.

### Professional roles and workforce implications

The redistribution of tasks may affect job satisfaction and perceived meaning in work – both essential elements of the Quadruple Aim framework. Further research is needed to assess the impact of MDTs on provider experience, including workload, interprofessional collaboration and the risk of burnout. These aspects of the MDT model will be explored in greater depth in our forthcoming research.

### Patient experience

The reorganization may also have implications for patient experience. The assignment of a named nurse and improved access pathways were designed to enhance continuity and patient trust. However, future studies should include patient-reported experience measures (PREMs) and outcomes (PROMs) to assess perceived accessibility, responsiveness and shared decision-making in MDT-based care.

### Strengths and limitations

The strengths of this study include its large absolute number of patients within a well-defined, population-based chronic disease cohort, inclusion of both primary and secondary care cost data, and a quasi-experimental design with controls. Unlike many RCTs, the study did not restrict the patient population; it used an unselected, population-based cohort, making it comprehensive in that respect. The use of routinely collected EHR data enhances the generalizability of the findings to real-world primary care settings. Nevertheless, because diagnoses were ascertained from routine registers during follow-up, descriptive morbidity measures reflect recorded case mix rather than true baseline morbidity. Differences in diagnostic intensity related to access and utilisation may therefore influence these measures. For this reason, diagnostic characteristics are not used to assess baseline comparability between intervention and control groups. Importantly, the study design is population-based, and there was no evidence of differential population inflow or outflow between intervention and control centres. The main analyses therefore rely on within-group changes over time and differences in cost and utilisation trajectories, rather than on comparisons of diagnostic case mix at baseline.

However, several additional limitations must be acknowledged. The implementation of the MDT model varied between centers, and follow-up time differed due to staggered roll-out. The different roll-out periods were considered in the analysis by comparing 2021–2023 but need to be recognized when drawing conclusions. Randomization was not feasible, but the Finnish catchment-area system helped balance baseline demographic and socio-economic differences.

Actual cost data was not possible to gather but the method of using the full-cost invoicing prices for foreigners has been used previously in similar comparisons. In Finland’s public healthcare, pricing is cost-based; profit margins are not included. Primary care costs in this study are based on standardized visit-level unit prices that incorporate all costs allocated to a patient contact under the local invoicing framework at the time the prices were defined. While this approach captures total visit-level costs, it does not allow disentanglement of changes in specific internal work components, such as increased physician consultative input within the MDT model. Consequently, the cost analyses should be interpreted as reflecting changes in service utilisation under a fixed cost-allocation structure, rather than detailed changes in workforce time use or task distribution.

In addition, the study does not include time-limited implementation costs related to the introduction of the MDT model, such as staff training or process development, as the focus of the analysis was on routine service delivery during the follow-up period rather than on the implementation phase itself. These limitations should be considered when interpreting primary care cost estimates, but they do not affect the analysis of secondary care costs, which are based on directly measured hospital expenditure data.

Clinical quality was assessed using only HbA1c as an outcome. Although the study population also included patients with hypertension and coronary heart disease, disease-specific clinical quality indicators for these conditions could not be analysed due to non-systematic recording and substantial missingness in routine EHR data. Analysing such variables would have introduced a high risk of measurement bias. Consequently, clinical quality assessment was intentionally restricted to outcomes that could be measured reliably and comparably across centres and over the full follow-up period. Although a statistically significant age difference was observed between groups, the absolute difference was small and was addressed through regression-based adjustment within a difference-in-differences framework. Propensity score matching was considered but not applied to preserve the population-based design and avoid loss of generalizability. The HbA1c analysis was based on a substantial and stable number of patients with repeated measurements in both groups, supporting the interpretation that the absence of deterioration reflects maintenance of long-term diabetes care quality rather than insufficient statistical power.

A limitation of this study is that intervention and control centres did not start from fully comparable baseline conditions in terms of access to non-urgent physician care. In the intervention centres, accumulated waiting lists were reduced prior to or during the implementation of the MDT model, whereas long waiting times persisted in several control centres throughout follow-up.

The approaches to backlog reduction differed between intervention centres. In one centre (Iso Omena), backlog reduction required a one-time, limited investment corresponding to approximately 20 physician working days. In another centre (Kilo), accumulated queues were addressed during the early phase of MDT implementation through reorganisation of team-based workflows without additional staffing resources. In the remaining intervention centres (Tapiola and Leppävaara), waiting lists were reduced prior to MDT implementation through internal measures without dedicated additional resources.

Consequently, part of the observed changes in utilisation and costs – particularly during the early phases of follow-up – may reflect transitions from backlog-clearing activity toward more stable service delivery in some intervention centres rather than steady-state effects of the MDT model alone. This heterogeneity in transition pathways should be considered when interpreting the difference-in-differences estimates for outcomes closely linked to access and utilisation.

Nevertheless, organisational changes related to the COVID-19 pandemic may have influenced patient mix and service use patterns in individual control centres during the early follow-up period. While this context should be acknowledged, it does not undermine the interpretation of the control group as representing persistently constrained access to primary care.

Because the intervention consisted of a multifaceted system-level reorganisation implemented over time, the analyses do not allow precise attribution of observed effects to individual components or to specific changes in post-intervention trends. The year-specific estimates reflect cumulative and persistent differences between groups following the transition phase rather than isolated dynamic slope changes. This should be considered when interpreting temporal patterns in the results. Also, because detailed high-frequency operational metrics were not consistently available across centres, we were unable to formally distinguish short-term level changes at implementation from changes in post-intervention trends. The results should therefore be interpreted as reflecting combined and cumulative effects of access restoration, workflow reorganisation and sustained MDT-based care delivery rather than isolated effects of individual components.

Finally, the lack of quality-of-life data precluded a full cost-effectiveness analysis.

## Conclusions

The reorganisation of primary care workflows using a MDT model was accompanied by a slower growth of secondary care costs and a modest reduction in visit-based primary care costs, while glycaemic control remained stable. The observed shift toward nurse-coordinated and remote consultations reflects changes in service delivery and resource allocation within primary care, while physician in-person visits decreased slightly.

Prior evaluations of team-based or integrated care typically examined single sectors or utilisation endpoints; few have reported combined primary and secondary costs together with clinical quality in the same analysis, and even fewer in a Nordic, cost-based pricing context. Using a population-based cohort, routine EHR data within a quasi-experimental framework, this study describes lower total per-patient cost levels in centres introducing the MDT model by the end of follow-up, alongside stable glycaemic control. The documented shift toward nurse-coordinated and remote contacts and slightly fewer physician in-person visits characterises differences in care organisation and utilisation patterns between settings.

## References

[1] Tynkkynen LK, Pulkki J, Tervonen-Gonçalves L, et al. Health system reforms and the needs of the ageing population—an analysis of recent policy paths and reform trends in Finland and Sweden. Eur J Ageing. 2022;19(2):221–232. doi: 10.1007/s10433-022-00699-x. 35465210 PMC9012246

[2] Stange KC, Miller WL, Etz RS. The role of primary care in improving population health. Milbank Q. 2023;101(S1):795–840. doi: 10.1111/1468-0009.12638. 37096603 PMC10126984

[3] THL. Finnish Institute for Health and Welfare. Access to treatment in primary health care. Finnish Institute for Health and Welfare (in Finnish); 2025 [Internet]. Available from: https://thl.fi/tilastot-ja-data/tilastot-aiheittain/terveyspalvelut/hoitoonpaasy-perusterveydenhuollossa

[4] Amnesty International. “Tiedän etten saa apua.” Terveydenhuollon eriarvoisuus Suomessa; 2023 [Internet]. Amnesty International. Available from: https://www.amnesty.fi/uploads/2023/06/terveydenhuollon-eriarvoisuus-suomessa_amnesty-international-suomen-osasto_06_2023.pdf

[5] Holster T, Nguyen L, Häkkinen U. The role of occupational health care in ambulatory health care in Finland. Nordic J Health Econ. 2022;10(1):61–81. doi: 10.5617/njhe.8561.

[6] Jokelin E, Piirainen L, Mustonen E, et al. Improving access, mixed continuity: effects of multidisciplinary teams on primary health-care in Finland – a quasi-experimental study. Scand J Prim Health Care. 2025;43(4):745–758. doi: 10.1080/02813432.2025.2502658. 40338140 PMC12632202

[7] Rocks S, Berntson D, Gil-Salmerón A, et al. Cost and effects of integrated care: a systematic literature review and meta-analysis. Eur J Health Econ. 2020;21(8):1211–1221. doi: 10.1007/s10198-020-01217-5. 32632820 PMC7561551

[8] Jokelin E, Karreinen S, Mustonen E, et al. Clinical and economic outcomes of multidisciplinary team members in primary care: a scoping review. BMC Health Serv Res. 2025;25(1):1025. doi: 10.1186/s12913-025-13243-1. 40764920 PMC12323019

[9] Carter R, Riverin B, Levesque JF, et al. The impact of primary care reform on health system performance in Canada: a systematic review. BMC Health Serv Res. 2016;16(1):324. doi: 10.1186/s12913-016-1571-7. 27475057 PMC4967507

[10] Moe J, Kirkland SW, Rawe E, et al. Effectiveness of interventions to decrease emergency department visits by adult frequent users: a systematic review. Acad Emerg Med. 2017;24(1):40–52. doi: 10.1111/acem.13060. 27473387

[11] Brainard JS, Ford JA, Steel N, et al. A systematic review of health service interventions to reduce use of unplanned health care in rural areas. J Eval Clin Pract. 2016;22(2):145–155. doi: 10.1111/jep.12470. 26507368

[12] Smith SM, Soubhi H, Fortin M, et al. Managing patients with multimorbidity: systematic review of interventions in primary care and community settings. BMJ. 2012;345(1):e5205. doi: 10.1136/bmj.e5205. 22945950 PMC3432635

[13] Jimenez G, Matchar D, Koh GCH, et al. Multicomponent interventions for enhancing primary care: a systematic review. Br J Gen Pract. 2021;71(702):e10–e21. doi: 10.3399/bjgp20X714199. 33257458 PMC7716873

[14] Sikka R, Morath JM, Leape L. The Quadruple Aim: care, health, cost and meaning in work. BMJ Qual Saf. 2015;24(10):608–610. doi: 10.1136/bmjqs-2015-004160. 26038586

[15] Kuo Y, Agrawal P, Chou L, et al. Assessing association between team structure and health outcome and cost by social network analysis. J Am Geriatr Soc. 2020;69(4):946–954. doi: 10.1111/jgs.16962. PMC8166955 33289067

[16] Dehmer SP, Baker-Goering MM, Maciosek MV, et al. Modeled health and economic impact of team-based care for hypertension. Am J Prev Med. 2016;50(5 Suppl. 1):S34–S44. doi: 10.1016/j.amepre.2016.01.027. 27102856 PMC8456755

[17] Meyers DJ, Chien AT, Nguyen KH, et al. Association of team-based primary care with health care utilization and costs among chronically ill patients. JAMA Intern Med. 2019;179(1):54–61. doi: 10.1001/jamainternmed.2018.5118. 30476951 PMC6583420

[18] Russo J. Cost-effectiveness of a multicondition collaborative care intervention: a randomized controlled trial. Arch Gen Psychiatry. 2012;69(5):506. doi: 10.1001/archgenpsychiatry.2011.1548. 22566583 PMC3840955

[19] Malmivaara A. Assessing validity of observational intervention studies – the benchmarking controlled trials. Ann Med. 2016;48(6):440–443. doi: 10.1080/07853890.2016.1186830. 27238631 PMC5152539

[20] Bice TW, Boxerman SB. A quantitative measure of continuity of care. Med Care. 1977;15(4):347–349. doi: 10.1097/00005650-197704000-00010. 859364

[21] Satokangas M, Arffman M, Koskela T, et al. Vältettävissä olevien sairaalahoitojaksojen mittari tukee perusterveydenhuollon suoriutumisen arviointia. Finn Med J. 2023;2023(78):e34914.

